# Reduced pulmonary blood flow in regions of injury 2 hours after acid aspiration in rats

**DOI:** 10.1186/s12871-015-0013-0

**Published:** 2015-03-18

**Authors:** Torsten Richter, Ralf Bergmann, Guido Musch, Jens Pietzsch, Thea Koch

**Affiliations:** 1 Department of Anesthesia and Intensive Care, Carl Gustav Carus University Hospital, Technische Universität Dresden, Dresden, Germany; 2 Department of Radiopharmaceutical and Chemical Biology, Institute of Radiopharmaceutical Cancer Research, Helmholtz-Zentrum Dresden- Rossendorf, Dresden, Germany; 3 Department of Anesthesia, Critical Care and Pain Medicine, Massachusetts General Hospital and Harvard Medical School, Boston, Massachusetts USA; 4 Department of Chemistry and Food Chemistry, Technische Universität Dresden, Dresden, Germany

**Keywords:** Acute lung injury, Respiratory aspiration, Positron emission tomography, Pulmonary circulation, Pulmonary perfusion, Adult respiratory distress syndrome

## Abstract

**Background:**

Aspiration-induced lung injury can decrease gas exchange and increase mortality. Acute lung injury following acid aspiration is characterized by elevated pulmonary blood flow (PBF) in damaged lung areas in the early inflammation stage. Knowledge of PBF patterns after acid aspiration is important for targeting intravenous treatments. We examined PBF in an experimental model at a later stage (2 hours after injury).

**Methods:**

Anesthetized Wistar-Unilever rats (n = 5) underwent unilateral endobronchial instillation of hydrochloric acid. The PBF distribution was compared between injured and uninjured sides and with that of untreated control animals (n = 6). Changes in lung density after injury were measured using computed tomography (CT). Regional PBF distribution was determined quantitatively in vivo 2 hours after acid instillation by measuring the concentration of [^68^Ga]-radiolabeled microspheres using positron emission tomography.

**Results:**

CT scans revealed increased lung density in areas of acid aspiration. Lung injury was accompanied by impaired gas exchange. Acid aspiration decreased the arterial pressure of oxygen from 157 mmHg [139;165] to 74 mmHg [67;86] at 20 minutes and tended toward restoration to 109 mmHg [69;114] at 110 minutes (*P* < 0.001). The PBF ratio of the middle region of the injured versus uninjured lungs of the aspiration group (0.86 [0.7;0.9], median [25%;75%]) was significantly lower than the PBF ratio in the left versus right lung of the control group (1.02 [1.0;1.05]; *P* = 0.016).

**Conclusions:**

The PBF pattern 2 hours after aspiration-induced lung injury showed a redistribution of PBF away from injured regions that was likely responsible for the partial recovery from hypoxemia over time. Treatments given intravenously 2 hours after acid-induced lung injury may not preferentially reach the injured lung regions, contrary to what occurs during the first hour of inflammation.

Please see related article: http://dx.doi.org/10.1186/s12871-015-0014-z.

## Background

Lung aspiration is a common complication at the induction of general anesthesia and in emergency medicine. Lung atelectasis, alveolar edema, peribronchial hemorrhage, and neutrophil migration are consequences of an aspiration event [1,2] and may lead to impaired gas exchange and extended hospital stays. In addition, acid aspiration is one of the common causes of the development of acute respiratory distress syndrome, with a mortality rate of approximately 40% [3-5].

To date, lung inflammation has been treated with antibiotics and anti-inflammatory drugs such as steroids [6], with the aim of attenuating the inflammatory response as early as possible. Based on the present data, steroid therapy is not recommended after acid aspiration [2]. Targeted drug delivery would be a step toward treating lung inflammatory processes more specifically. Experimental interventions to treat aspiration have included the use of a hyperosmolar solution [7], a hypertonic saline solution [8], pentoxifylline [9], concentrated human albumin and furosemide [10]. These treatments were administered intravenously and, therefore, their delivery to injured areas was dependent on regional pulmonary perfusion. Consequently, knowledge of the blood flow pattern in the lungs after an aspiration event is critical for targeting the delivery of treatments to areas where they are most needed.

The distribution of pulmonary blood flow (PBF) increased in the injured lung regions of rats 10 minutes after the administration of hydrochloric acid (HCl) [11], and this PBF distribution pattern remained within the first hour after acid aspiration [12], which was within the time frame of the first peak of the inflammatory response in the rats [13]. Furthermore, the lung weight constantly increased within the first two hours after aspiration [14], indicating edema formation due to changes in lung microvascular permeability [10].

The manner by which the PBF distribution changes as an ensuing inflammatory process evolves has not been established. Therefore, we non-invasively characterized the spatial PBF distribution in rats 2 hours after acid-induced lung injury using [^68^Ga]-radiolabeled microspheres and small animal positron emission tomography (PET). The focus of this study was on evaluating the PBF distribution pattern in areas of lung injury in a later stage of acid-induced acute lung injury. PBF in these areas was specifically compared with PBF in uninjured areas and in control animals. The clinical relevance of this study lies in its answer to the question of whether, in this stage of aspiration-induced lung injury, damaged areas will still be preferentially perfused.

## Methods

### Experimental setting

All of the experiments were conducted using 11 male Wistar-Unilever rats (mean body weight, 355 g; range 339–381 g) according to the German Regulations for Animal Welfare. The protocol was approved by the local Ethics Committee for Animal Experiments (Landesdirektion Dresden, 24D-9168.11-4/2007-1).

In each experiment, rats were anesthetized using a combination of desflurane (7-12%) and a single intraperitoneal injection of ketamine (75 mg/kg). Local anesthesia during the operation was provided by infiltration with 1% lidocaine. Body temperature and supine position were maintained by placing the animals in a custom-built, heated, plastic tube. Catheters were inserted into the femoral artery and femoral vein for the following purposes: to measure arterial blood pressure and heart rate (Model 54S, Component Monitoring System; Hewlett-Packard, Saronno, Italy), to obtain arterial blood samples for blood gas analyses (ABL 50, Radiometer, Copenhagen, Denmark), and to provide intravenous infusions. To administer HCl to the lung, we inserted a cannula after tracheostomy.

### Experimental protocol

The anesthetized, spontaneously breathing animals were allowed to stabilize after preparation and were then randomly assigned to the control group (n = 6) or the injury group (n = 5), and baseline values were recorded. In both groups, a catheter was blindly inserted to the maximum depth through the tracheostomy tube into either the left or right lung. Only in the injury group was acid aspiration induced by unilateral intra-bronchial administration of 0.1 M HCl (0.4 mL/kg body weight) through the catheter. The amount of HCl administered into the supine-positioned animals was limited to prevent life-threatening lung failure and spillover to the contralateral lung. The control group received nothing through the catheter.

**Figure 1:**
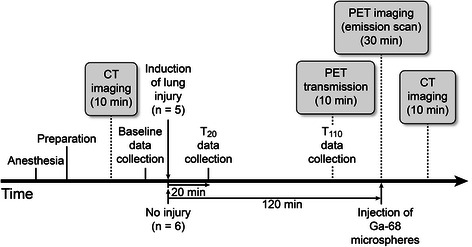
**Study protocol schema.**

Physiological parameters (including blood gases and hemodynamics) were determined at baseline (T_0_) and then 20 (T_20_) and 110 (T_110_) minutes after acid aspiration or baseline measurements of the injury and control group, respectively. Computed tomography (CT) was used to identify regions of increased pulmonary density after aspiration. Spatial PBF was quantitatively determined using radiolabeled microspheres and PET. Figure 1 shows the protocol schema.

### Small animal computed tomography imaging

The degree of injury was assessed using lung density measurements obtained from 10-minute CT scans of the lung, which were acquired at baseline (T_0_) and at T_110_ using a micro-CT scanner (Skyscan 1178 micro-CT system, Kontich, Belgium) with a resolution of 0.083 mm and the following specifications: 83-μm pixel size, 360-degree scanning, average of 2 frames, 85-mm field of view, 9 minute 7 second scanning time, 65-kV voltage, and 615-μA current. The data were exported and converted to the ECAT2 format.

### Radiolabeling of microspheres

Human serum albumin microspheres with a mean diameter of 20 μm were labeled with gallium-68 (^68^Ga), as previously described [15], to obtain injectable ^68^Ga-human serum albumin microspheres (Ga-68 microspheres).

### Small animal positron emission tomography imaging

A microPET® P4 scanner (Siemens Preclinical Solutions, Knoxville, TN, USA) with a spatial resolution of 1.85 mm full-width half maximum in the center of the field of view along both the tangential and radial directions was used. Following a 10-minute transmission scan, a 1-minute bolus infusion of Ga-68 microspheres was initiated, and a 30-minute emission scan was performed. Emission scans were corrected for decay and attenuation, and the frames were reconstructed using ordered subset expectation maximization. The voxel size was 0.8 × 0.8 × 1.2 mm^3^.

### Image processing

#### Registration

Image files were processed using the *ROVER* software (ABX GmbH, Radeberg, Germany). CT scans were imported in Dicom format and converted to the same voxel size as the PET scans. Residual differences between images were corrected using the *ROVER* registration module to obtain the same alignment for all 3 dimensions of the PET and CT images. The first and second CT scans of each animal were analyzed for changes in lung density. Poorly aerated and non-aerated areas, which were visually identified, were assumed to be areas of lung injury [16].

#### ROI definition

**Figure 2:**
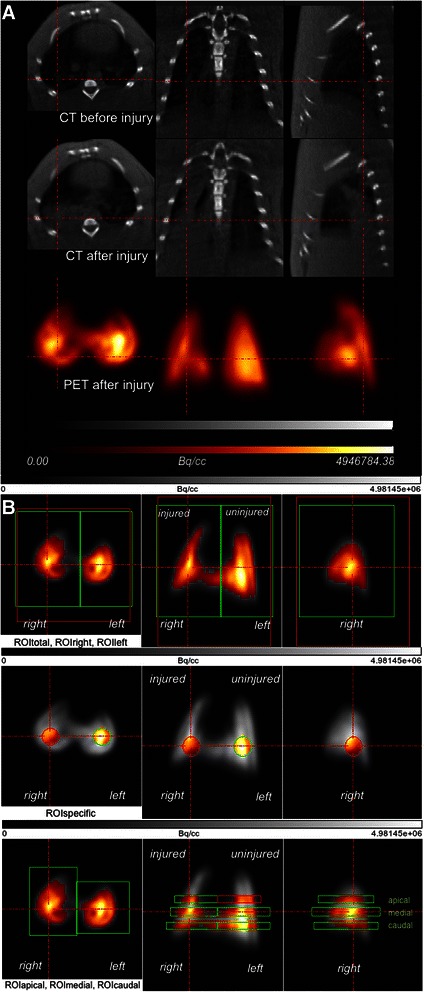
**Example of image processing after co-registration. (A)** Co-registered PET and CT images in the transverse, coronal, and sagittal planes of one animal; the CT image before and after injury and the corresponding PET scan image are shown. Dark areas in the CT scan represent aerated zones. The areas of high density were formed in the right lung after injury (red lines). The PET scan shows the distribution of PBF in the left and right lungs. **(B)** PET images and definition of the ROI for one lung in the transverse, coronal, and sagittal planes. The tracer activity in the lung is indicated in grayscale, from lowest (black) to highest activity (white). ROI masks were used for the threshold analysis and to calculate the median maximum standardized uptake values (SUV_max_) for each side of the lung in a 3-dimensional environment. The thresholds in the ROI masks for the entire lung (red: ROI_total_), the left and right lung (green), the spherical masks (ROI_specific_) (0.24 cm^3^) and the 6 ROIs (apical, medial, and caudal) are indicated by the highlighted regions of the color-coded areas. Color coding shows a relative scale in which the measurements are normalized to their mean values and represent regional tracer concentrations of Ga-68 microspheres from high (white) to low (black), as determined by PET in Bq/cm^3^. Red lines are focused on the area of injury.

Masks that described the volume for the regions of interest (ROIs) were created and placed over the co-registered PET scan of each animal. The ROI corresponding to the entire lung field on the PET image (ROI_total_) was divided into 2 sections along the sagittal plane, resulting in 2 separate ROIs (ROI_lung side_) for the right (ROI_right_) and left (ROI_left_) lungs (Figure 2). Masks were created to define transverse layers as representative subregions of the apical, middle, and caudal regions of the lung (Figure 2). The resulting ROIs (ROI_apical_, ROI_middle_, ROI_caudal_) were identically placed over the co-registered PET scan of each animal to compare the PET measurements between the apical, middle, and caudal regions of each lung. A spherical ROI mask was symmetrically placed on each lung side (between the middle and dorsal third of the sagittal axis, the centric and the lateral third at the transversal axis and the middle and caudal region along the vertical axis). Empirically, this standard position included the most frequently damaged lung regions after acid-induced lung injury, as observed previously in this aspiration model [11]. Areas of increased lung density following aspiration were identified on the CT scan and could be marked with this spherical ROI. The spherical ROI mask was identically placed over all co-registered CT and PET scans to define the ROI_specific_ (0.24 cm^3^), which was used to assess the ratios of the mean lung density in the injured versus non-injured lungs from the CT scan and to determine the PBF from the PET scan (Figure 2).

### Pulmonary blood flow

The PBF distribution was derived by analyzing the pulmonary distribution of Ga-68 microspheres within the ROIs [15]. When using this method, regional perfusion is proportional to the number of microspheres that reach the ROI. Regional PBF is quantified as the fractional flow to the ROI relative to the ROI_total_. Regional PBF was analyzed from the selected ROI (ROI_lung side_, ROI_specific_, ROI_apical_, ROI_middle_, ROI_caudal_) (Figure 2) for each lung in the control and injury groups. The maximum standardized uptake value (SUV_max_) was calculated [11] with respect to the ^68^Ga half-life within the ROIs to quantitatively compare PBF patterns. The PBF in each region was analyzed as the ratio of PBF on the injured side to that on the uninjured side in the aspiration group and as the ratio of PBF on the left side to that on the right side in the control animals.

### Statistical analysis

All of the data are expressed as median values and 25^th^ and 75^th^ percentiles (median [25-75%]) unless otherwise indicated.

Changes over time were analyzed globally using the Friedman test for each time series, followed by a paired *t*-test for individual comparisons with baseline. An unpaired *t*-test with Welch’s correction was used to compare the 2 groups with respect to hemodynamic values and SUVs at individual time points. A nonparametric Wilcoxon 2-tailed, matched-pairs signed rank test was used for data comparison within groups. Calculations were performed using GraphPad Prism 5.02 for Windows (GraphPad Software Inc., San Diego, CA, USA). Statistical significance was set at *P* < 0.05.

## Results

### Physiological variables

Global physiological data are shown in Table 1. The physiological variables did not differ between the control and aspiration groups at baseline and did not change significantly during the study period in the control animals. Acid aspiration worsened pulmonary gas exchange, decreasing arterial partial pressure of oxygen (PaO_2_) and arterial oxygen saturation (SpaO_2_) values; the greatest impairment occurred after 20 minutes, and the values tended toward recovery at 110 minutes. In the aspiration group, the mean arterial pressure decreased from 99 mmHg [85;102] to 90 mmHg [83;91] at 20 minutes (T_20_) (*P* = 0.06) and to 83 mmHg [69;87] at 110 minutes (T_110_) (*P* = 0.04).

**Table 1:** **Physiological variables**

	T_0_	T_20_	T_110_
**PaO**_2_ **, mmHg**			
Control	152 [152;160]	148 [141;170]	124 [111;163]
Injury	157 [139;165]	74 [67;86]^a,b^	109 [69;114]^a^
**PaCO**_2_ **, mmHg**			
Control	53 [51;61]	54 [53;59]	54 [53;64]
Injury	59 [56;62]	57 [50;63]	47 [43;68]
**pH**			
Control	7.36 [7.33;7.37]	7.35 [7.33;7.35]	7.35 [7.29;7.36]
Injury	7.31 [7.26;7.36]	7.46 [7.3;7.46]	7.31 [7.27;7.39]
**SpaO**_2_ **, %**			
Control	99.3 [99.3;99.4]	99.3 [99.2;99.4]	98.8 [98.3;99.3]
Injury	99.3 [99.1;99.5]	94.5 [92.8;96.4]^a,b^	98.2 [93.1;98.4]
**HR, beats/min**			
Control	294 [276;324]	319 [292;373]	306 [295;339]
Injury	293 [256;326]	303 [278;326]	299 [273;331]
**MAP, mmHg**			
Control	93 [91;102]	100 [90;102]	95 [86;98]
Injury	99 [85;102]	90 [83;91]	83 [69;87]^b^
**RR, breaths/min**			
Control	59 [58;63]	59 [56;62]	61 [55;64]
Injury	57 [53;66]	60 [58;71]	63 [61;66]
**T, °C**			
Control	35.6 [35.2;35.9]	35.7 [34.8;36]	36 [35;36.8]
Injury	35.1 [34.4;35.4]	35.1 [34.7;36]	36 [35.2;36.6]

Physiological variables at certain time points in the control group (n = 6) and injury group (n = 5). Values represent the median [interquartile range]. HR, heart rate; MAP, mean arterial blood pressure; PaO_2_, arterial oxygen tension; PaCO_2_, arterial carbon dioxide tension; pH, arterial pH; RR, respiratory rate; SpaO_2_, arterial oxygen saturation; T, body temperature; T_0_, baseline; T_20_, 20 minutes after acid aspiration (injury group) or after baseline (control group); T_110_, 110 minutes after acid aspiration (injury group) or after baseline (control group). ^a^*P* < 0.05 vs. T_0_; ^b^*P* < 0.05, Control vs. Injury.

### Density changes

An increase in CT-derived lung density (comparing the CT scan at baseline with the CT scan after injury) allowed localization of the effects of acid administration on the lung tissue. In the injury group, ROI_specific_ analysis revealed localized increased density in the left (n = 4) or right (n = 1) lung. The ratio of the mean lung density in the injured versus non-injured lungs (1.4 [1.2-1.5]) was significantly higher than the ratio of lung density in the left versus right lungs in the control group (1.1 [1.0-1.1], *P* = 0.006).

### Regional pulmonary blood flow

**Figure 3:**
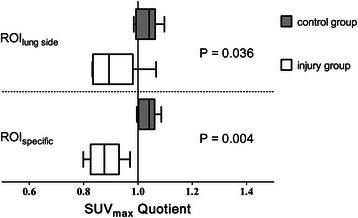
**PBF ratios for ROI**_lung__side_**and ROI**_specific_**.** PBF ratio expressed as the median of the quotients of the maximum standardized uptake value (SUV_max_) in the left versus right side in the control group (n = 6) and injured versus uninjured side in the acid aspiration group (n = 5) for each lung (ROI_lung__side_) and for a spherical region of interest of 0.24 cm^3^ (ROI_specific_). The data are shown as the median [25th and 75th percentiles] and the minimum to maximum values. Lower mean concentrations of Ga-68 microspheres in the injured region yield a PBF ratio < 1 in ROI_lung side_ or ROI_specific_. *P* compared with the control group.

**Figure 4:**
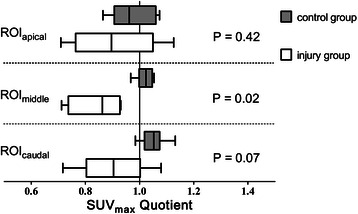
**PBF ratios for ROI**_apical_**, ROI**_middle_**and ROI**_caudal_**.** Maximum standardized uptake value (SUVmax) ratio for the apical, middle, and caudal ROIs, expressed as the median of the quotients of SUVmax left/right side in the control group (n = 6) and the median of the quotients of SUVmax injured/uninjured side in the injury group (n = 5). The data are shown as the median [25th and 75th percentiles] and the minimum to maximum values. A PBF quotient < 1 was determined based on lower concentrations of Ga-68 microspheres in the injured lung. *P* compared with the control group.

**Figure 5:**
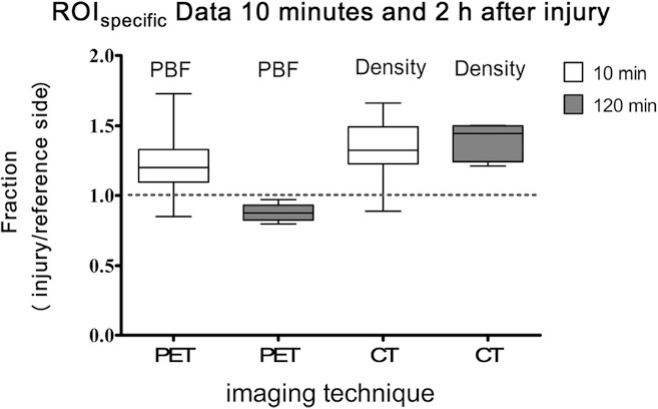
**PBF and density ratios for ROI**_specific_**.** Data from PET and CT images representing the PBF and density fraction of injured to uninjured lung for a region of interest (ROI_specific_) in all of the injured animals in the present study (2 hours after injury; n = 5) and a previous study (n = 12) [11] in which the spatial PBF distribution was measured 10 minutes after acid aspiration injury. The data are shown as the median [25th and 75th percentiles] and the minimum to maximum values. The ROI_specific_ was spherical and of identical size (0.24 cm^3^) for all animals and for the PET and CT images of each animal. For the mean density values (CT images) and for the mean radioactivity concentration values (PET images), the presented fractions were calculated using the quotient of the ROI_specific_ values for the injured side versus that of the non-injured side of each animal. A greater mean density on the injured side resulted in a CT quotient > 1 in the ROI_specific_. Lower mean concentrations of Ga-68 microspheres on the injured side 2 hours after injury yielded a PET quotient < 1 in the ROI_specific_, whereas higher mean concentrations of Ga-68 microspheres on the injured side 10 minutes after injury yielded a PET quotient > 1 in the ROI_specific_.

Table 2 shows the PBF data for the following regions: the entire lung field (ROI_total_), right lung, left lung, ROI_specific_, and 3 transverse planes (apical, middle, and caudal) of the injured and uninjured lungs. The PBF ratios in the injured versus uninjured lungs of the aspiration group were significantly lower than those in the left versus right lungs of the control group when PBF was referenced to the entire lung (ROI_lung side_), ROI_specific_ (Figure 3), and ROI_middle_ (Figure 4). The PBF ratios of ROI_specific_ from the current study and a previous study [11] are shown in Figure 5.

**Table 2:** **Pulmonary blood flow values for all regions of interest**

	Control group		Injury group
**Entire lung**	111 [107;115]		121 [108;137]
**ROI**_lung side_			
Left side	110 [107;115]	Injured side	101 [94;129]
	*P* = 0.16		*P* = 0.125
Right side	106 [103;111]	Uninjured side	117 [108;135]
**ROI**_specific_			
Left side	110 [107;113]	Injured side	98 [92;123]
	*P* = 0.06		*P* = 0.06
Right side	106 [104;109]	Uninjured side	117 [107;135]
**ROI**_apical_			
Left side	92 [90;100]	Injured side	95 [71;113]
	*P* = 0.56		*P* = 0.31
Right side	98 [93;101]	Uninjured side	97 [92;115]
**ROI**_middle_			
Left side	107 [104;111]	Injured side	96 [83;119]
	*P* = 0.22		*P* = 0.06
Right side	104 [103;108]	Uninjured side	114 [108;133]
**ROI**_caudal_			
Left side	110 [105;115]	Injured side	101 [94;116]
	*P* = 0.06		*P* = 0.19
Right side	104 [100;111]	Uninjured side	112 [103;132]

Pulmonary blood flow expressed as maximum standardized uptake values in the injury group (n = 5) and control group (n = 6) for each ROI. Values represent the median [interquartile range]. *P* values were calculated to compare each ROI of the left and right lung within the control group and between the injured and contralateral lungs within the acid aspiration group.

## Discussion

Using small animal imaging techniques, we quantitatively demonstrated the effects of acid aspiration on PBF in anesthetized, spontaneously breathing rats 2 hours after injury. Lung injury increased the density of the damaged regions and was accompanied by impairments in physiological variables. The regional response 2 hours after acid aspiration consisted of decreased PBF in injured lung regions accompanied by attenuation of hypoxemia and residual hypotension.

### Acid aspiration

Acute lung injury was induced with a small amount of HCl, which is associated with a low mortality rate [17]. While the physiological parameters remained stable in the control animals, acid-induced lung injury caused deterioration of PaO_2_ and SpaO_2_ values shortly after the administration of HCl. These parameters recovered to values that were not significantly different from those of the control group at 110 minutes after injury; however, PaO_2_ did not reach baseline levels. In line with previous observations [11], acid injury induced hypotension. Although hypoxemia improved over time, the arterial blood pressure did not. The heart rate remained unchanged during the observation period, which was consistent with findings reported in the literature [9,11].

Contact with concentrated acid forms membrane pores in the alveolar epithelium and initiates a sequence of events that results in the recruitment of leukocytes and the formation of edema [18]. Within 4 hours after administration, HCl caused cellular injury, which has been characterized by an increased number of intra-alveolar erythrocytes, the recruitment of neutrophils, and fibrin formation [13,19]. The impact of acid on lung tissue is represented by an increase in tissue density in the injured areas [20,9]. Using CT scans to detect lung density changes [21], we found that almost all of the animals were injured in the middle and caudal areas, which was anticipated based on bronchial anatomy. The density ratio (injury/reference side of ROI_specific_) obtained 2.5 hours after injury in this study (1.4 [1.2-1.5]) was comparable to the density ratio observed within 1 hour after acid administration in rats (1.3 [1.2;1.5]) in a previous study [11] (Figure 5).

### PBF

Data regarding the effects of acid aspiration on regional PBF are limited [11,22]. We observed different PBF ratios between the injury and control groups that demonstrated decreased PBF on the injured side compared with the non-injured side in the aspiration group. This difference was the result of changes on either side, namely, a non-significant increase in PBF on the non-injured side and a non-significant decrease in PBF on the injured side (Table 2). Additionally, these findings indicate a redistribution of lung perfusion away from injured regions over time. The location of this redistribution differs from that observed within the first hour after aspiration [11,12]. In fact, contrary to the results of the present study, the very early phase (10 minutes after acid aspiration injury in rats) was characterized by a redistribution of perfusion toward regions of increased density, with higher PBF ratios between the injured and the uninjured lung sides in ROI_specific_ in the aspiration group compared with the control group (Figure 5) [11].

When comparing 3 transverse ROIs, the PBF was different in areas where the HCl produced the most abundant density changes, namely, the middle region. Again, the uninjured side showed a non-significantly higher PBF than the injured side, which resulted in a significantly different PBF ratio in the ROI_middle_ in the injured animals compared with the control group (Figure 4). This finding is contrary to observations from the early stage of acid-induced injury, where the PBF ratio was different for the apical, middle, and caudal ROIs because of increased PBF on the injured side [11].

Multiple factors may be responsible for the reduction of PBF in injured regions during the later stages of aspiration-induced ALI. The spatial distribution of PBF is dependent on oxygen tension [23], and experimental hypoxia can cause changes in flow distribution [24-26]. The hypoxic pulmonary vasoconstriction may have been blunted or blocked by the acid injury [27] and may have been further limited by relaxing factors that were released from the endothelium in the pulmonary vessels during hypoxia [28] in the early stages of the inflammatory response (10 minutes after acid aspiration). Those factors may have been compensated for over time, resulting in hypoxic pulmonary vasoconstriction 2 hours after injury. Otherwise, the initial hyperperfusion in injured regions may have led to additional alveolar damage [29]. Therefore, it can be speculated that the increased PBF in aspiration areas could promote a loss of fluid through the damaged endothelium with subsequent regional edema. Additionally, the stepwise development of alveolar hemorrhage, intra-alveolar edema, and interstitial edema [30,31] after acid administration in rats is an inflammatory response that depends on circulating mediators [13,32,33]. The edema formation, with resulting extravascular pressure in the lung parenchyma, may reduce the spatial PBF in damaged areas over time.

Pulmonary perfusion distribution has been examined in different models of acute lung injury and has been reported to change based on position [34-37] or interventions in the ventilator setting [38,37,39]. To study the changes in PBF in the aspiration model used in this study, intra-individual comparisons of PBF are possible.

Beginning from the first minute after acid aspiration in spontaneously breathing rats, damaged regions are preferentially perfused within the first hour after injury [11,12]. The fact that the PBF decreases as the inflammatory process continues shows that regional hyperperfusion in regions of acid induced lung damage is limited over time. Therefore, interventions such as targeted drug delivery do not preferentially reach areas of lung injury after 2 hours in this model of acute lung injury. However, it remains unclear whether the PBF distribution pattern in this model is relevant to other types of lung injury or to other species.

Lung injury such as acid aspiration-induced injury, blunt trauma/lung contusion and bacterial inflammation, which can occur in isolated parts of the lung, may show completely different perfusion distribution in a certain time frame, depending on the degree and onset of increased intravascular permeability, interstitial edema, neutrophilic alveolitis [19] and the therapeutic interventions such as ventilation. Therefore, the PBF distribution pattern requires further investigation in different animal models and over time.

### Limitations of the study

In this model of anesthetized, spontaneously breathing rats, we considered the effects of acid alone, in the absence of particulate matter, which is commonly a component of aspirated fluid in humans. In humans, aspiration frequently affects the lower right pulmonary region; however, the fact that the left side was most affected in this study may represent aspiration events that can occur with a spatial alignment different from the supine or right position. This study focused on the PBF distribution between the first and second peaks of the inflammatory response in rats [13]. Potentially underlying mechanisms for the PBF pattern, such as endothelial damage, cardiac output, edema formation, platelet aggregation, and changes in regional ventilation, were not considered.

The number of animals used in the current study when assessing differences in the SUV_max_ ratios was small but with an adequate power for the statements made (especially for ROI_specific_ and ROI_middle_). To better reflect clinical experience, further studies are needed to obtain information about the PBF distribution pattern in cases in which greater aspiration involves a larger volume of injured lung. Additionally, the possible need for ventilatory support in cases of massive aspiration necessitates studies examining the influence of positive pressure ventilation (in different settings) on PBF. Interestingly, a higher proportion of spontaneous breathing reduced lung injury as compared with protective mechanical ventilation in a surfactant depletion model [40] or after acid aspiration [41]. In the current study, the PET images represent the PBF during the entire breathing cycle in spontaneously breathing animals. The breathing pattern was continuously monitored based on the respiratory rate only, because depression of ventilation in spontaneously breathing rats was recognized to be due to a depression in the respiratory rate, as discussed previously [15]. Therefore, the absence of changes in the respiratory rate suggests stable respiration during the experiment.

The standardized acid aspiration model and the PET technique used in the present study have been previously discussed [11,15]. We used a 30-minute data acquisition time for the emission scan to optimize the activity count rate; the influence of potential density changes during the PET scan on PBF, obtained from volume data, has been shown to not significantly influence the results [11].

## Conclusions

In this experimental rat model of acid aspiration-induced lung injury, we observed a decrease in PBF in injured lung regions compared with uninjured regions 2 hours after the aspiration event. This redistribution of pulmonary perfusion away from injured regions likely accounted for the partial recovery from hypoxemia that was observed at 110 minutes compared with 20 minutes after injury. Taken together, the results of the present and previous studies [11,12], which covered the time window of 2 hours post aspiration, suggest that an initial shift of perfusion toward injured regions after aspiration is followed by redistribution away from them. The potential clinical implication of these experimental findings is that intravenous treatments may not preferentially reach injured lung regions unless they are administered within a specific time during the first hour of the aspiration event.

## Abbreviations

ALI: Acute lung injury
CT: Computed tomography
HCl: Hydrochloric acid
PaO_2_: Arterial oxygen tension
PBF: Pulmonary blood flow
PET: Positron emission tomography
ROI: Region of interest
SpaO_2_: Arterial oxygen saturation
SUV: Standardized uptake value
